# Efficient lasing in size-tunable self-assembled organic microcavities

**DOI:** 10.1039/d6sc03498e

**Published:** 2026-07-23

**Authors:** Lulu Xue, Hongyan Shi, Jiafan Qu, Huachun Deng, Ziyang Chen, Jiaxin Du, Bo Gao

**Affiliations:** a Institute of Modern Optics, School of Physics, Harbin Institute of Technology Harbin 150001 China gaobo@hit.edu.cn; b Qianyuan Laboratory Hangzhou 310000 China; c Key Laboratory of Micro-Nano Optical-acoustic-electronic System of Ministry of Industry and Information Technology Harbin 150001 China; d Key Laboratory of Micro-Optics and Photonic Technology of Heilongjiang Province, Harbin Institute of Technology Harbin 150001 China; e Harbin Institute of Technology Shenzhen China; f Collaborative Innovation Center of Extreme Optics, Shanxi University Taiyuan Shanxi 030006 China

## Abstract

Blue-emitting organic semiconductor microcavities are attractive candidates for low-threshold, narrow-linewidth microlasers, but the controllable fabrication of high-quality cavities remains challenging. As a readily accessible organic gain material, 1,4-bis(4-methylstyryl)benzene (*p*-MSB) can spontaneously form single-crystalline microcavities through solution self-assembly and exhibits favorable amplified spontaneous emission (ASE) characteristics. However, self-assembled *p*-MSB microcavities typically adopt elongated hexagonal geometries with unequal side lengths, leading to increased optical mode leakage, weakened optical confinement, and limited lasing performance. Here, by regulating the solution atmosphere during self-assembled crystal growth, we obtained high-quality *p*-MSB hexagonal microcavities. The regular *p*-MSB hexagonal microcavities (regular-PHMs) with nearly identical side lengths were obtained by further adjusting the *p*-MSB concentration. As the cavity geometry approaches an ideal regular hexagon, optical mode leakage is effectively suppressed, leading to efficient lasing with a low optical pumping threshold of ∼4.12 µJ cm^−2^, a narrow linewidth of 0.2 nm, and a high-quality factor of ∼2283. In addition, single-mode lasing was realized by reducing the size of the regular-PHMs. These excellent lasing properties are closely associated with the cavity geometry and size, both of which can be precisely controlled through the crystal preparation process. This work demonstrates that size-tunable organic microcavities provide a promising platform for optical circuits and next-generation miniaturized optoelectronic devices.

## Abstract 2

By regulating the self-assembly process, regular *p*-MSB hexagonal microcavities with tunable sizes are obtained and exhibit high-*Q*, distinct sharp peak emission and single-mode lasing (left picture).

## Graphical abstract

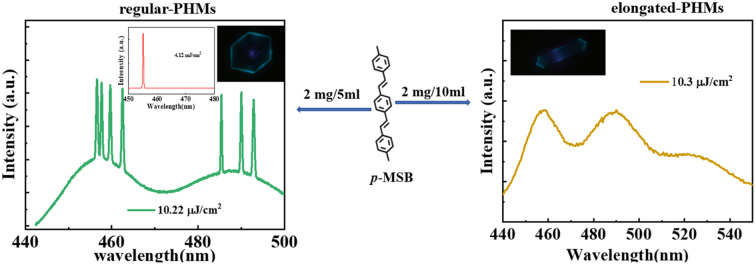

## 1 Introduction

Compared with inorganic semiconductors, organic semiconductor materials exhibit distinctive optoelectronic characteristics and have attracted considerable attention as promising candidates for laser and lab-on-a-chip (LoC) photonic devices.^1–5^ Organic gain materials used for laser applications mainly include organic crystals, small organic molecules, dendritic starburst molecules, and conjugated polymers.^3,6,7^ Over the past few decades, substantial efforts have been devoted to reducing the lasing threshold of organic semiconductor lasers through both material engineering and device-structure optimization. Among these material systems, organic crystals are particularly attractive because of their anisotropic optical and electrical properties, well-defined crystal structures, ease of synthesis and processing, structural tunability, and broad wavelength tunability across the visible spectral range.^5^ In addition, their ability to form self-assembled optical resonators provides unique advantages for semiconductor lasers applications. In particular, organic single-crystalline micro/nanostructures, which possess highly ordered molecular packing, low defect density, and grain-boundary-free architectures, can support efficient photon propagation and charge transport.^8–10^ Moreover, the ordered molecular arrangement in organic single crystals often leads to anisotropic charge-transport behavior, as demonstrated in organic field-effect transistors.^11,12^ Owing to their intrinsically superior carrier-transport properties relative to many organic materials,^13^ organic single crystals are considered the most promising candidates for electrically pumped organic lasers.^14^ As a gain structure, the shape of organic single crystals is of great significance. It can affect the gain size of micro–nano devices, thereby influencing the light excitation threshold.^15^ However, because the carrier mobility of organic semiconductors is significantly lower than that of inorganic semiconductors, the realization of electrically pumped and continuous-wave (CW) organic lasers remains highly challenging.^16^

To improve lasing performance and reduce the excitation threshold, the design of optical resonators has become a central issue in organic microlasers. Among various cavity architectures, whispering gallery mode (WGM) optical micro- and nanocavities have attracted extensive attention because of their high fluorescence quantum efficiency, strong light confinement, and a low amplified spontaneous emission (ASE) threshold.^3,17^ In WGM resonators, light circulates along the cavity boundary *via* repeated total internal reflection, endowing these systems with low optical loss and small mode volume.^18,19^ These characteristics make organic WGM cavities highly promising for miniaturized photonic integration and for the development of low-threshold, electrically pumped, and continuous-wave organic lasers. To realize high-quality WGM organic resonators, a variety of microstructures have been explored, including spheres,^20–22^ circular disks,^23^ hexagonal disks,^19,24,25^ and rectangular microplates.^18,26^ Among the reported gain materials, *p*-distyrylbenzene (DSB) derivatives have been widely investigated because of their high crystal quality and excellent ASE performance.^27–29^ Low-temperature photoluminescence (PL) studies have revealed pronounced vibronic structures, high photoluminescence quantum yields, and self-waveguide-induced laser action at room temperature.^27^ In addition, molecular modification, such as introducing flexible alkoxy/alkyl chains or altering substitution positions on the benzene rings, has proven effective in improving optical performance, suppressing fluorescence quenching, and lowering excitation thresholds.^30–32^ For example, Kabe *et al.* developed two DSB derivatives, 1,4-bis(4-methylstyryl) benzene (*p*-MSB) and 1,4-bis(2-methylstyryl) benzene (*o*-MSB), and demonstrated an ultra-low ASE threshold of 14 µJ cm^−2^ in *o*-MSB single crystals, which was markedly lower than that of DSB based systems.^29^ Fu *et al.* further observed optically pumped lasing action in rectangular *o*-MSB morphology microplate resonators, with a high *Q* of ∼1500 and a low threshold of ∼540 nJ cm^−2^.^26^ In contrast, no lasing emission was observed in elongated hexagonal *p*-MSB microplates, mainly because the elongated hexagonal morphology supports low-*Q* resonant modes and weak optical confinement. These results indicate that the morphology of microstructures plays a decisive role in the resonator characteristics and lasing performance of organic microcavities. Further calculations suggested that regular hexagonal microplates with the same side length could provide much better optical confinement and support optically pumped lasing with high *Q* factors as high as ∼2500.^26^ These findings highlight the decisive role of crystal morphology in regulating the resonator characteristics and lasing performance of organic microcavities.

The fundamental optical and ASE characteristics of 1,4-bis(4-methylstyryl)benzene (*p*-MSB) have been investigated for different molecular morphologies. However, previous studies mainly focused on numerical simulations, while the controllable growth of well-defined organic microstructures remains a considerable challenge. In this work, we demonstrate the controllable preparation of *p*-MSB single-crystalline hexagonal microcavities and systematically investigate their morphology- and size-dependent optical and lasing properties. By regulating the solution atmosphere (carbon disulfide, CS_2_) during the self-assembly process, high-quality *p*-MSB single crystals with improved structural regularity were obtained. At low *p*-MSB concentration, elongated hexagonal microcavities could be fabricated. In particular, regular hexagonal microcavities (regular-PHMs) with nearly identical side lengths were successfully achieved by increasing the concentration of *p*-MSB organic molecules in carbon disulfide (CS_2_), which exhibit much better optical confinement than elongated microcavities. Owing to the improved cavity geometry, the regular hexagonal microcavities show efficient lasing with a low threshold of ∼4.12 µJ cm^−2^ and a high-quality factor of ∼2283. More significantly, single-mode lasing was successfully achieved by future reducing the crystal size. These results demonstrate that the crystal quality and cavity geometry can be controlled through the self-assembled growth atmosphere, while the crystal size can be tuned by solution concentration, providing an effective route toward high-performance organic microcavity lasers for integrated photonic circuits.

## 2 Results and discussion

**Fig. 1:**
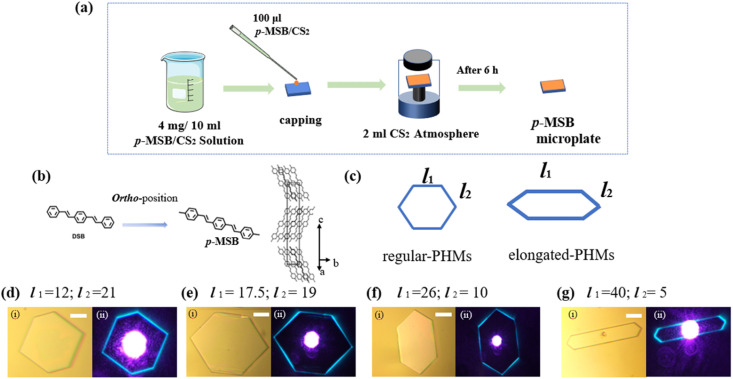
Growth and the bright optical image of the PHMs. (a) The schematic illustration of the growth process of *p*-MSB microcrystals. (b) The chemical structure of the model isomeric π-conjugated organic molecule 1,4-bis (4-methylstyryl) benzene (*p*-MSB).^29^ (c) Schematic models of two types of the *p*-MSB microplates. Here, *l*_1_ and *l*_2_ denote the long edge and the short edge lengths of the hexagonal microplate, respectively. The aspect ratio *η* is defined as *l*_1_/*l*_2_. Microplates with *η* > 1 are named elongated hexagonal microplates (elongated-PHMs), whereas those with *η* ∼1 are named regular hexagonal single crystal microplates (regular-PHMs). (d–g) Bright optical image (i) of a representative PHM in a small scale. (ii) The fluorescence microscopy image of the as-prepared PHMs excited at 400 nm. Scale bar is 10 µm.

*p*-MSB hexagonal microcavities (PHMs) with different morphologies were grown by a self-assembly method under the solution atmosphere of carbondisulfide (CS_2_) as schematically illustrated in Fig. 1(a) (details of materials are provided in the SI S1). By regulating the CS_2_ solution atmosphere during the self-assembly process, high-quality *p*-MSB single crystals with improved structural regularity were obtained. Fig. 1(b) shows the chemical structure of the model isomeric π-conjugated small organic molecule 1,4-bis (4-methylstyryl) benzene (*p*-MSB), a simplified derivative of *p*-distyrylbenzene (DSB). The crystallographic data and structure refinement information and photographs are summarized and shown in Table S1.^29^ This nearly planar structure promotes strong supramolecular interactions, resulting in a layer-by-layer molecular arrangement in a typical herringbone (H-aggregate) packing motif, laterally extending along the *ab*-plane, while the long *c*-axis (about 4 nm) is parallel to the crystal *z*-axis.^29^ In our experiment, 2 mg and 4 mg of *p*-MSB were completely dissolved in 10 mL of CS_2_ at room temperature, and then the resulting solutions were dropped onto the quartz substrate for 6 h, respectively.

By controlling the concentration of organic molecules, single crystals with different sizes can be obtained. As shown in Fig. 1(c), the PHMs can be classified into two types: regular hexagonal single-crystalline microcavities (regular-PHMs), in which all side lengths are nearly identical, and elongated hexagonal microcavities (elongated-PHMs), in which the side lengths are unequal. Here, *l*_1_ and *l*_2_ represent the long edge and the short edge lengths of the hexagonal microcavities, respectively. The aspect ratio *η* is defined as *l*_1_/*l*_2_. For the regular-PHMs, the *η* is close to 1, whereas for elongated-PHMs, *η* is larger than 1. Previous theoretical and experimental studies have suggested that the intrinsic growth morphology of *p*-MSB crystals prepared by a conventional solution-drying method tends to be an elongated hexagonal plate-like structure.^26,29^ Consistent with this prediction, the bright optical image of as-prepared PHMs revealed predominantly elongated hexagonal morphology in Fig. S2. Notably, by adding the molecular concentration, the regular-PHMs could be obtained, indicating that the molecular concentration plays an important role in regulating the crystal morphology of these self-assembled microstructures. The representative bright-field optical image (i) and fluorescence microscopy image (ii) of regular-PHMs and elongated-PHMs are shown in Fig. 1(d–g). Under excitation with a 400 nm femtosecond laser, both types of microcavities exhibit intense pale-blue emission and clear optical waveguiding characteristics. As observed in Fig. 1(d and e), the fluorescence images show bright pale-blue edges and a relatively dark interior region, indicating strong optical confinement within the cavity. According to the atomic force microscopy (AFM) results shown in Fig. S3, the regular PHMs have a thickness of approximately 500–600 nm. In contrast, Fig. 1(f) and (g) show the microscopy fluorescence image of the elongated-PHMs and exhibit intense pale-blue emission with waveguiding characteristics. The flatness of the facets and the elongated hexagonal shape of PHMs are clearly shown in the AFM image and it is confirmed that they have the height (*h*) of ∼400–500 nm. These results demonstrate that the growth atmosphere plays an important role in improving crystal quality and structural regularity, while the molecular concentration mainly governs the crystal size and also influences the final cavity morphology.

**Fig. 2:**
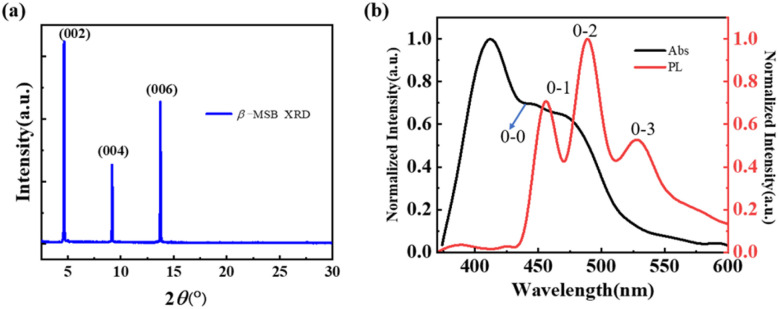
The characterization of *p*-MSB microcrystals. (a) X-ray diffraction (XRD) pattern of the as-grown *p*-MSB microcrystals. The pattern reveals the single crystalline nature and the orthorhombic phase. (b) Room-temperature absorbance (black curve) and photoluminescence (PL) spectra (red curve) of *p*-MSB microcrystals.

The crystalline quality and phase structure of the as-grown *p*-MSB microcrystals were examined by X-ray diffraction (XRD), as shown in Fig. 2(a). The exclusive observation of (002)-related diffraction peaks indicates a highly preferred orientation of the *p*-MSB microcrystals with well-defined layered stacking along the *c*-axis (Fig. 1(b)). Previous studies have suggested that the thermodynamically stable phase of *p*-MSB microcrystals at room temperature is orthorhombic, in which the molecules are arranged layer by layer in the typical herringbone (H-aggregate) packing motif.^29^ Our results reveal that all the reflections can be attributed to the (002), (004) and (006) crystal planes, wherein the sole existence of the (00*h*) series diffraction peaks not only reflects the single crystalline nature but also confirms the orthorhombic crystal structure of the microcrystals, in good agreement with previous studies. Fig. 2(b) depicts the room-temperature absorption and photoluminescence (PL) spectra of the single microcrystal with high fluorescence quantum efficiency ∼ of 88.1% measured on a quartz substrate (setups shown in Fig. S4 in SI III). A broad absorption peak with a maximum at 425 nm, assigned to the 0–0 transition, is observed in the absorption spectrum (black curve), which is characteristic of the H-aggregate nature of the *p*-MSB microcrystals. The PL spectrum (red curve) exhibits three emission peaks at 458 nm, 488 nm and 530 nm, as shown in Fig. S4(f). Following the conventional assignments reported for *p*-MSB, these bands are denoted as the 0–1, 0–2, and 0–3 vibronic emission bands, respectively (Fig. S4(g)).^26,29^ Here, these labels are used phenomenologically and do not imply a harmonic progression involving a single, uniquely identified normal mode. Furthermore, the polarization-dependent PL measurements (SI, Fig. S4(e)) of both microcavities oscillate with a period of 180°, indicating pronounced optical anisotropy originating from the ordered molecular packing in the single crystals. These results confirm that the as-grown *p*-MSB microcrystals possess high crystalline quality, a well-defined orthorhombic structure, and anisotropic optical properties, providing a solid basis for their application as high-performance organic microcavity laser resonators.

**Fig. 3:**
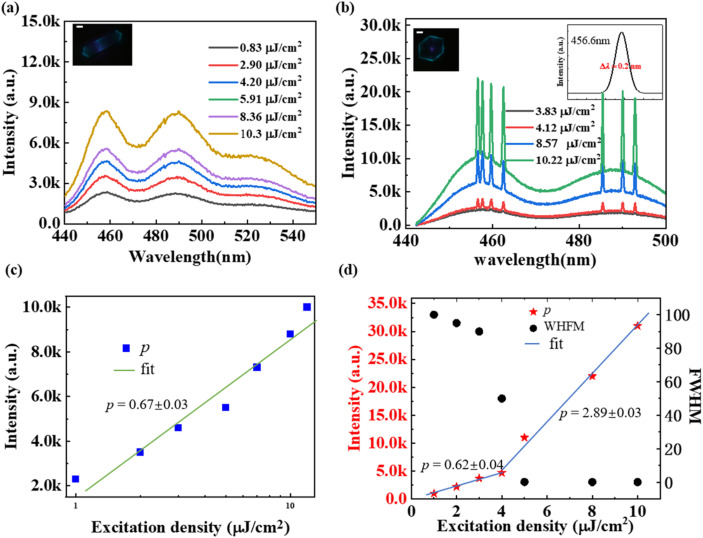
Pump-dependent photoluminescence and lasing behavior of elongated and regular *p*-MSB microplates. (a) Room-temperature PL spectra of an elongated-PHM recorded under different pump fluences ranging from 0.83–10.3 µJ cm^−2^. Left inset: the fluorescence microscopy image of the elongated-PHM. Scale bar is 10 µm. (b) Room-temperature PL spectra of one regular-PHM recorded under different pump fluences ranging from 3.8–10.2 µJ cm^−2^. Left inset: the fluorescence microscopy image of this regular-PHM. Scale bar is 10 µm. Right inset: enlarged view of the emission peak in the 455–463 nm range, showing a minimum FWHM, Δ*λ*, ∼0.20 nm. The corresponding maximum quality factor (*Q*) is 2283, calculated from *λ*/Δ*λ* = 456.6 nm/0.20 nm. (c) Integrated PL intensity of the 0–1 emission band (∼460 nm) as a function of the pump densities for the elongated-PHM. (d) Red symbols: integrated PL intensity (corresponding to the left axis) of the 0–1 emission as a function of the pump densities for a regular-PHM, together with the corresponding power-law fit (blue line). Black symbols: the FWHM (corresponding to the right axis) of PL (0–1 emission) as a function of the pump densities for a regular-PHM.

At room temperature, only spontaneous fluorescence emission was observed from the single elongated *p*-MSB hexagonal microcavity due to the low *Q* limitation. The pump-dependent PL spectra of the representative elongated-PHM on the pump density is shown in Fig. 3(a) (setup in Fig. S5). As the pump fluence increases from 0.83 to 10.3 µJ cm^−2^, the PL intensity increases gradually. However, no lasing action is detected at either 0–1 or 0–2 peaks throughout the entire excitation range, until the morphology of the hexagonal-PHM suffered damage under the increasing optical excitation. Consistent with this result, the integrated intensity of 0–1 and 0–2 emission peak as a function of excitation intensity in Fig. 3(c) follows a sublinear power-law dependence, *I* ∝ *x*^*p*^ with *p* = 0.67 ± 0.03. This sublinear behavior can be attributed to bimolecular quenching, namely exciton–exciton annihilation.^33,34^ These results indicate that the elongated PHM does not provide sufficient optical feedback to support lasing. In a previous report, the ASE spectrum of *p*-MSB single crystals exhibited two sharp peaks at 456 and 488 nm.^29^ Clear lasing behavior was achieved in the individual regular *p*-MSB microcavity, which possesses a high *Q*. Fig. 3(b) shows the pump-dependent PL spectra of one regular-PHM. Distinct sharp peaks emerge on top of the 0–1 and 0–2 transition emission bands, revealing the onset of laser oscillation. In a hexagonal cavity composed of six sides, six-bounce whispering-gallery-like modes are generally expected to dominate the resonant behavior. At a relatively low pump density of 3.83 µJ cm^−2^, the PL spectrum is dominated by broad spontaneous emission (the black curve). Below the lasing threshold, the integral intensity shows a sublinear relationship with pump fluence, with *p* = 0.62 ± 0.04 (Fig. 3(d)), again suggesting the presence of exciton–exciton annihilation. When the pump density increases from 4.12 to 10.22 µJ cm^−2^, the emission intensity increases sharply and enters a superliner regime with *p* = 2.89 ± 0.03, indicating the onset of lasing behavior. Accordingly, the threshold can be clearly observed at 4.12 µJ cm^−2^ and the fluorescence microscopy images with low and high threshold are shown in Fig. S6. Above the threshold, the fluorescence intensity concentrates at the six corners and form lasing.

To further investigate the excited-state dynamics associated with lasing, pump–probe transient absorption microscopy was employed (Time-Tech Spectra, Co., Ltd, SI S7). At a very low excitation density of 0.3 *E*_th_ at 456.6 nm, the transient decay time constant of the individual PHM follows single-exponential function, with a decay time >5 ns, which is limited by accessible long-time window of our apparatus. Above the threshold, for example at 1.5 *E*_th_, the transient decay time constant becomes bimolecular, with a short decay time of 9.62 ± 0.74 ps at 456.6 nm, which is attributed to stimulated emission. The decay time always collapses to <10 ps. The pronounced shortening of the decay time above threshold provides further evidence for lasing in the regular-PHM. The right inset of Fig. 3(b) presents the zoomed spectrum around 456.6 nm, from which the minimum full width at half maximum (FWHM) is determined to be as small as 0.20 nm. Due to unavoidable variations in size and morphology during the self-assembly process, individual microcavities are not perfectly identical. Slight shifts in the lasing peak wavelength (454.2–459 nm) are observed among different samples. The cavity quality factor, defined as *Q* = *λ*/Δ*λ*,^27^ is estimated to be 2283 ± 12 obtained from above 20 individual microcavities. Such a high *Q* value indicates the formation of a high-quality resonant mode within the regular-PHMs. This indicates that the high-*Q* resonant mode is formed within the regular *p*-MSB single-crystalline resonators. Taken together, these results strongly suggest that the markedly improved lasing performance of the regular-PHMs, compared with the elongated-PHMs, originates from their more favorable cavity geometry and stronger optical confinement.

**Fig. 4:**
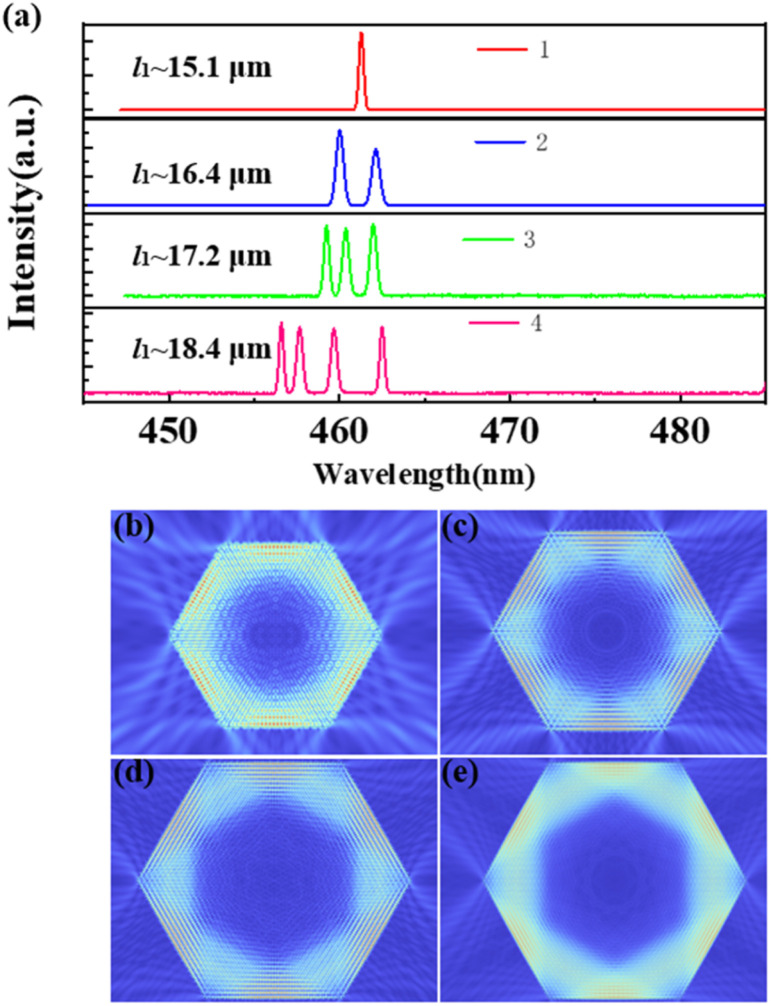
Size-dependent lasing behavior of regular *p*-MSB hexagonal microcavities. (a) Laser spectra of four regular-PHMs with different sizes (*l*_1_ = 15.1, 16.4, 17.2, and 18.4 µm). (b–e) Simulated two-dimensional normalized electric-field (*λ* = 458 nm, *n* = 1.80) in the corresponding microcavity resonators. Regular-PHMs with different sizes can all support efficient WGM optical resonators for lasing oscillators. Red corresponds to the highest field density, and blue indicates the lowest field density.

The regular-PHMs were found to support size-dependent lasing behavior, including both multimode and single-mode laser emission. In general, single-mode lasing has its inherent merits as excellent monochrome properties of the laser beam, fine optical coherence, and good stability of laser oscillation due to the absence of mode competition.^35^ In principle, lasing behavior is determined by the spectral overlap between the cavity resonances and the gain profile of the material.^38^ Therefore, smaller resonators are more likely to exhibit single-mode lasing because they support fewer resonant states within the gain bandwidth. To clarify the size-dependent of lasing behavior in these organic single-crystalline microcavities, spatially resolved spectra were collected from regular PHMs with different lateral sizes. Fig. 4(a) presents the lasing spectra sequentially of four representative regular-PHMs, with *l*_1_ varying from 15.1 µm to 18.4 µm. It should be noted that the lasing spectra are collected from the whole microcavity. Under sufficiently high optical pumping above the lasing threshold, such as 2*E*_th_, the stimulated emission becomes the dominant contribution, as evidenced by the emergence of sharp lasing peaks and the pronounced narrowing of the emission line width, while the broad spontaneous-emission background remains relatively weak. As a result, the measured spectra are mainly composed of sharp lasing peaks. Notably, in a small regular PHM with *l*_1_ ∼ 15.1 µm, single-mode lasing was successfully achieved at room temperature. This result indicates that the single-crystalline nature of these microcavities contributes to the high density of optical gain, which can account for the observed laser action within the regular-PHM with small size. To gain more insight into the lasing behaviors in these microcavities, numerical simulations were carried out using a finite element method (FEM). Fig. 4(b–e) show the simulated electromagnetic distribution of the four-corresponding regular-PHM resonators. The hexagonal pattern suggests that the optical mode is reflected by the side facets and is well-supported in the cavity. The field intensity at six corners is relatively weaker due to light leakage, in good agreement with previous reports.^36,37^ These simulation results indicate that regular-PHMs with different sizes can all act as efficient WGM optical resonators for lasing oscillators.

The lasing mode as a function of side length *l* was further investigated for the hexagonal cavities in Fig. S8(a). PHMs with comparable thicknesses (450 ± 10 nm) and different lengths were selected to minimize the effect of thickness difference. As the side length increases from 15.9 to 18.9 µm, the distance between two adjacent modes (Δ*λ*_m_) decreases from 3.71 to 0.72 nm, as shown in Fig. S8(b). The extracted Δ*λ*_m_ values are inversely proportional to the cavity size, which is consistent with the expected behavior of 6-WGM lasing in hexagonal resonators.^24^ In a hexagonal WGM cavity, the free spectral range (Δ*λ*_m_) is calculated using:^27^
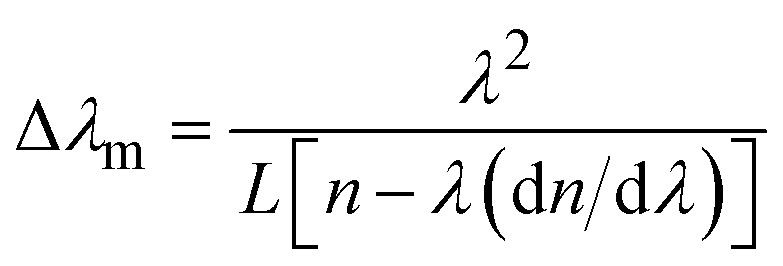

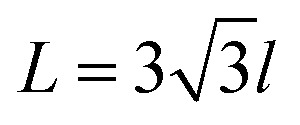
where *λ* is the resonance wavelength, *L* is the round-trip distance length and *l* is the edge length of the regular-PHM.^27^ Accordingly, *n* is the refractive index ∼1.80. This nonlinear relationship in Fig. S8(b) agrees well with the optical transmission path of the WGM mode in the hexagonal cavity. These results demonstrate that regular *p*-MSB hexagonal microcavities with different sizes can all sustain efficient WGM lasing, while reducing the cavity size effectively decreases the mode density and facilitates the realization of single-mode laser emission.

**Fig. 5:**
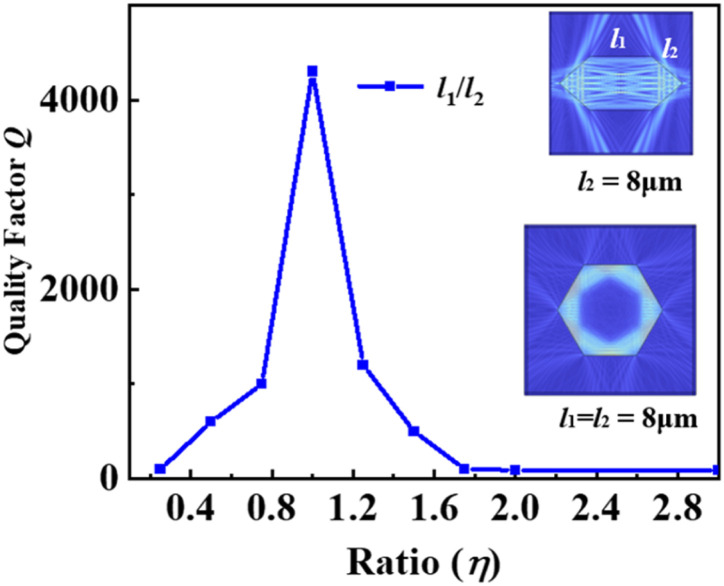
Dependence of the cavity quality factor on the side-length ratio of the hexagonal microplate. The aspect ratio *η* is defined as *l*_1_/*l*_2_, where *l*_1_ and *l*_2_ denote the long and short edge lengths of the hexagonal microplate, respectively, while *l*_1_ + *l*_2_ is fixed at 16 µm. Top inset: simulated two-dimensional normalized electric-field (surface: electric field norm (V m^−1^), *λ* = 458 nm, *n* = 1.80) of elongated *p*-MSB hexagonal microplates corresponding to the *η* = 2. Bottom inset: simulated 2D normalized electric-field (surface: electric field norm (V m^−1^), *λ* = 458 nm, *n* = 1.80) of regular *p*-MSB hexagonal microplates with *η* = 1. Red and blue represent the highest and lowest electric-field intensities, respectively.

To further understand the cavity-mode behaviour in hexagonal single-crystalline microcavities, we simulated the electromagnetic field distribution of hexagonal cavities with different side length ratios, as shown in Fig. 5. In the model, *l*_1_ and *l*_2_ represent the long edge and the short edge lengths of the hexagonal microcavities, respectively. The aspect ratio *η* is defined as *l*_1_/*l*_2_, while the sum of *l*_1_ + *l*_2_ is kept constant at 16 µm. The calculated *Q* as a function of *η* is plotted in Fig. 5. The *Q* factor increases dramatically with the increasing value of *η* (*η* < 1), and then decreases dramatically with the increasing value of *η* (1 < *η* < 2). When *η* = 1, the resonator is the regular hexagonal resonator and the *Q* value reaches its maximum value ∼4300 with excellent light confinement in the bottom inset. The absolute *Q* values may be overestimated because out-of-plane radiation loss, finite-thickness effects, surface roughness, and substrate coupling are not included. In this case, the electric field is well confined inside the cavity, as shown in the bottom inset, indicating excellent optical confinement and low radiation loss. By contrast, when the cavity deviates from the regular hexagonal geometry and becomes elongated (*η* > 1), the quality factor decreases markedly. As shown in the top inset for *η* = 2, a substantial portion of the electric field leaks out of the cavity, especially near the short side facets. This pronounced field leakage indicates that the elongated hexagonal cavity cannot efficiently confine the optical mode, resulting in significantly increased radiative loss. This pattern with large field leakage from four short side facets confirms our assumption and can well account for the poor lasing performance of the elongated-PHMs in Fig. 3(a). In Fig. S9, the quality factor *Q* of these regular hexagonal geometries is the largest when the side length >10 µm, which promises the efficient optical resonator for the laser operation. Due to out-of-plane radiation loss, surface roughness, finite-thickness effects, and possible substrate coupling, the experimental *Q* factors are lower than the simulated values. Nevertheless, the 2D FEM simulations remain useful for explaining the geometry-dependent trend in optical confinement.^26^ Therefore, these simulation results clearly demonstrate that the regular hexagonal geometry is much more favorable for sustaining high-*Q* resonant modes than the elongated geometry. The much better confinement in the regular-PHM is consistent with its high *Q* factor and efficient lasing behavior.

## 3 Conclusions

In summary, regular and elongated hexagonal *p*-MSB microcavities were successfully prepared through self-assembled crystal growth under controlled solution conditions. Impressively, we systematically investigated the whispering-gallery-mode (WGM) lasing behavior of these organic optical microresonators and the fluorescence resonance phenomenon was observed in the individual regular *p*-MSB hexagonal microcavity (regular-PHM) rather than the elongated hexagonal *p*-MSB microcavity (elongated-PHM). The regular-PHMs exhibit a much higher quality factor (*Q*) of ∼2283 and better optically pumped lasing performance above a lasing threshold of ∼4.12 µJ cm^−2^. The enhanced lasing performance in regular-PHMs is attributed to their stronger optical light confinement, which is further supported through numerical simulations. In addition, the lasing mode number of regular-PHMs decreases with the decreasing cavity size, and single-mode lasing was successfully realized in small regular-PHMs with an edge length of 9.5 µm. These self-assembled organic microcavities with tuneable morphologies and size represent promising micro-/nanoscale light sources for integrated photonic circuits.

## 4 Experimental section

### 4.1 Synthesis of PHMs

The *p*-MSB microcrystals with different morphologies were grown by a self-assembly method under the solution atmosphere of CS_2_ as schematically illustrated in Fig. S1. Specifically, 4 mg and 2 mg of *p*-MSB were dissolved in 10 mL of CS_2_ to prepare the *p*-MSB/CS_2_ solution, respectively. Then, 100 µL of the solution was drop-cast onto the substrate and capped. Afterward, the sample was kept in a 2 mL CS_2_ atmosphere for 6 h. Finally, the *p*-MSB microplate was obtained.

### 4.2 Lasing and time-resolved photoluminescence spectroscopy

Individual microplates were investigated at room temperature in air using a home-made optical microscope equipped with a 100 × 0.9 NA excitation objective. The second harmonic (400 nm, 150 fs, 1 kHz) of a regenerative amplifier (Spitfire, Spectra Physics) seeded with a mode-locked Ti: sapphire laser (Tsunami, Spectra Physics) was focused to a 50 µm-diameter spot to excite the selected individual microplates. The excitation beam from a femtosecond laser was linearly polarized. At the lasing threshold, the single-pulse energy was approximately 0.1 nJ, corresponding to a peak power of approximately 667 W. A linear polarizer was placed between the laser and the sample, and the excitation polarization angle was varied by rotating this polarizer. Then PL spectra were collected underneath by using a 100 × 0.9NA objective that was mounted on a 3D movable stage. A 450 nm long-wave pass dielectric filter was used to block any scattered excitation light. Finally, the collected PL was detected using a liquid-nitrogen-cooled CCD attached to a polychromator (Spectropro-550i, Acton). The photoluminescence spectra were collected using a spectrometer equipped with a CCD detector and a 1200 lines/mm grating. The spectral resolution was approximately 0.1 nm.

The transient dynamics were measured using microscale pump–probe spectroscopy. The effective temporal resolution at a probe wavelength of 460 nm, estimated from the time-zero coherent response of a blank glass substrate, was approximately 0.14 ps.

#### 4.2.1 Numerical simulation

The two-dimensional normalized electric-field distributions of the hexagonal microplate resonators with different morphologies were calculated using the finite element method (FEM). The refractive index of the PHMs was set to *n* = 1.80 at 458 nm according to previous reports.^26,29^ The electromagnetic eigenmodes were obtained by solving the source-free Maxwell eigenvalue problem using an eigenfrequency solver. No external excitation source was introduced in the eigenmode calculation.

A two-dimensional in-plane model was adopted, in which the simulated *x*–*y* plane corresponds to the experimentally observed microcavity plane (Fig. S9). Perfectly matched layers (PMLs) were applied at the outer boundaries of the computational domain to suppress artificial reflections. The computational domain was extended to twice the characteristic lateral dimension of the microcavity.

## Author contributions

The experimental work was conducted and the original draft was prepared by Lulu Xue. The TAS data were obtained by Lulu Xue and Jiafan Qu. The numerical simulations were performed by Huachun Deng and Ziyang Chen. The conception was proposed by Hongyan Shi and Bo Gao. All authors read and approved the final manuscript.

## Conflicts of interest

There are no conflicts to declare.

## Supplementary Material

SC-OLF-D6SC03498E-s001

## Data Availability

Data supporting the findings of this study are available from the corresponding author upon reasonable request. All relevant data are within the manuscript and the supplementary information (SI). Supplementary information: the preparation method and characterizations of *p*-MSB microcavities, optical path setup. See DOI: https://doi.org/10.1039/d6sc03498e.
