# Supplementary material for: Efficient lasing in size-tunable self-assembled organic microcavities
Source: Chem Sci. 2026 Jul 23;17(37):17789–96. doi: 10.1039/d6sc03498e (PMC13435121; doi:10.1039/d6sc03498e)
Supplement: Supplementary file 1 [file SC-017-D6SC03498E-s001.pdf]

Supplementary Information (SI) for Chemical Science.
This journal is © The Royal Society of Chemistry 2026

Efficient Lasing in Size-Tunable Self-Assembled Organic Microcavities

Lulu Xue,[a,c,d] Hongyan Shi,[b]* Jiafan Qu[a,c,d], Huachun Deng,[e] Ziyang Chen[a,c,d], Jiaxin Du[a,c,d] and Bo Gao[a,c,d,f] *

[a]Institute of Modern Optics, School of Physics, Harbin Institute of Technology, Harbin 150001, China

[b]Qianyuan Laboratory, Hangzhou 310000, China

[c]Key Laboratory of Micro-Nano Optical-acoustic-electronic System of Ministry of Industry and Information Technology, Harbin 150001, China

[d]Key Laboratory of Micro-Optics and Photonic Technology of Heilongjiang Province, Harbin Institute of Technology, Harbin 150001, China

[e]Harbin Institute of Technology, Shenzhen

[f]Collaborative Innovation Center of Extreme Optics, Shanxi University, Taiyuan, Shanxi 030006, China

*Author to whom correspondence should be addressed: shi.hong.yan@hit.edu.cn and gaobo@hit.edu.cn

S1 details in the experimental section

Materials

The compounds of 1,4-bis (4-methylstyryl) benzene (*p*-MSB) were purchased from Sigma-Aldrich and was used without further treatment. The solvent of carbondisulfide (CS_2, HPLC grade), was purchased from Beijing Chemical Agent Ltd., China, and was used without further treatment.

(a)

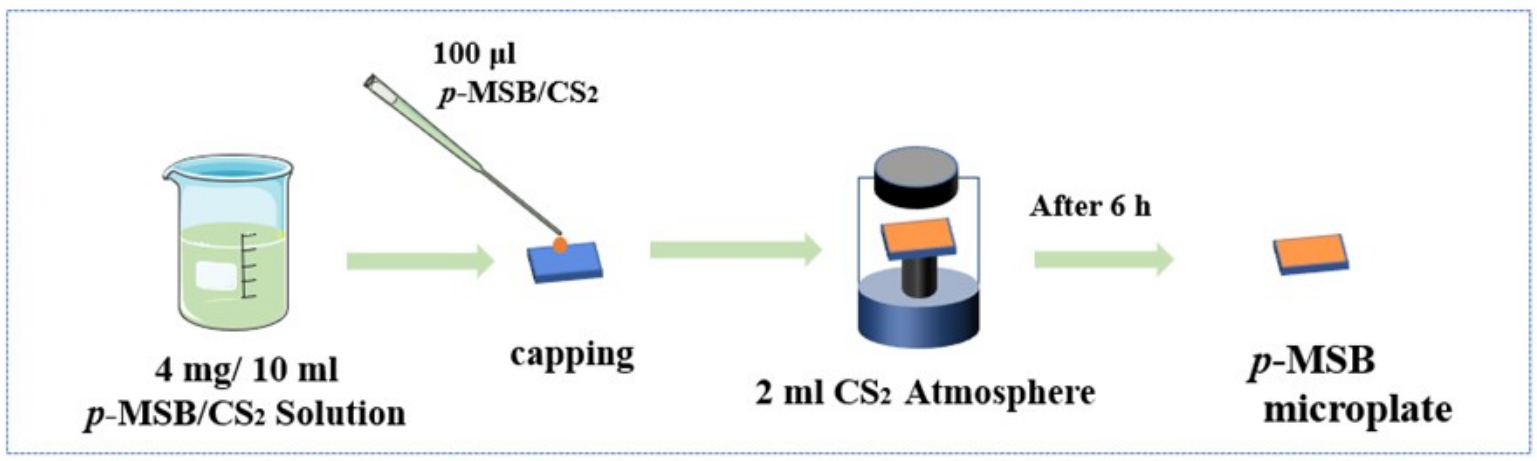

Figure S1 The schematic illustration of the growth process of *p*-MSB microcrystals.

The *p*-MSB microcrystals with different morphologies were grown by a self-assembled method under the solution atmosphere of carbondisulfide (CS_2) as schematically illustrated in Figure S1. Specifically, 4 mg and 2mg of *p*-MSB was dissolved in 10 mL of CS_2 to prepare the p-MSB/CS_2 solution, respectively. Then, 100 μL of the solution was drop-cast onto the substrate and capped. Afterward, the sample was maintained in a 2 mL CS_2 atmosphere for 6 h. Finally, the *p*-MSB microplate was obtained.

Structural characterization of microplates:

The X-ray diffraction (XRD) patterns were measured by a D/max 2400 X-ray diffractometer with Cu Kα radiation (λ = 1.54050 Å) operated in the 2θ range from 5° to 30°, by using the samples on the silicon substrate.

Fluorescence images were recorded using an Olympus research inverted system microscope (Olympus, X71) equipped with a charge couple device camera. The excitation source is the second harmonic (pulse width 150 fs) of a Ti:sapphire regenerative amplifier to a 40 μm diameter spot with a Gaussian beam profile. The samples were prepared by placing a drop of solution onto a cleaned quartz plate, and then the solvent evaporated.

S2 The bright optical image of the PHMs in a large scale

We have performed a statistical analysis of the p-MSB microcrystals obtained at concentrations of 2 and 4 mg per 10 mL CS_2 in Figure S2. For each concentration, crystals collected from three independently prepared samples were examined using optical microscopy. Figure S2 (a-c) show the optical microscopy image of *p*-MSB microcrystals obtained at concentrations of 2 mg per 10 mL CS_2. more than 150 crystals were analyzed, among which > 99 % were classified as elongated hexagonal microcavities. Figure S2 (d-f) show optical microscopy image of the *p*-MSB microcrystals obtained at concentrations of 4 mg per 10 mL CS_2. We can observe that the hexagonal single crystals are uniformly dispersed on the substrate. more than 150 crystals were examined, of which 42~47% were regular hexagonal microcavities (η~1). These statistical results confirm that increasing the *p*-MSB concentration from 2 to 4 mg per 10 mL CS_2 substantially increases the proportion of regular hexagonal microcavities, demonstrating that the crystal morphology can be effectively regulated by changing the solution concentration.

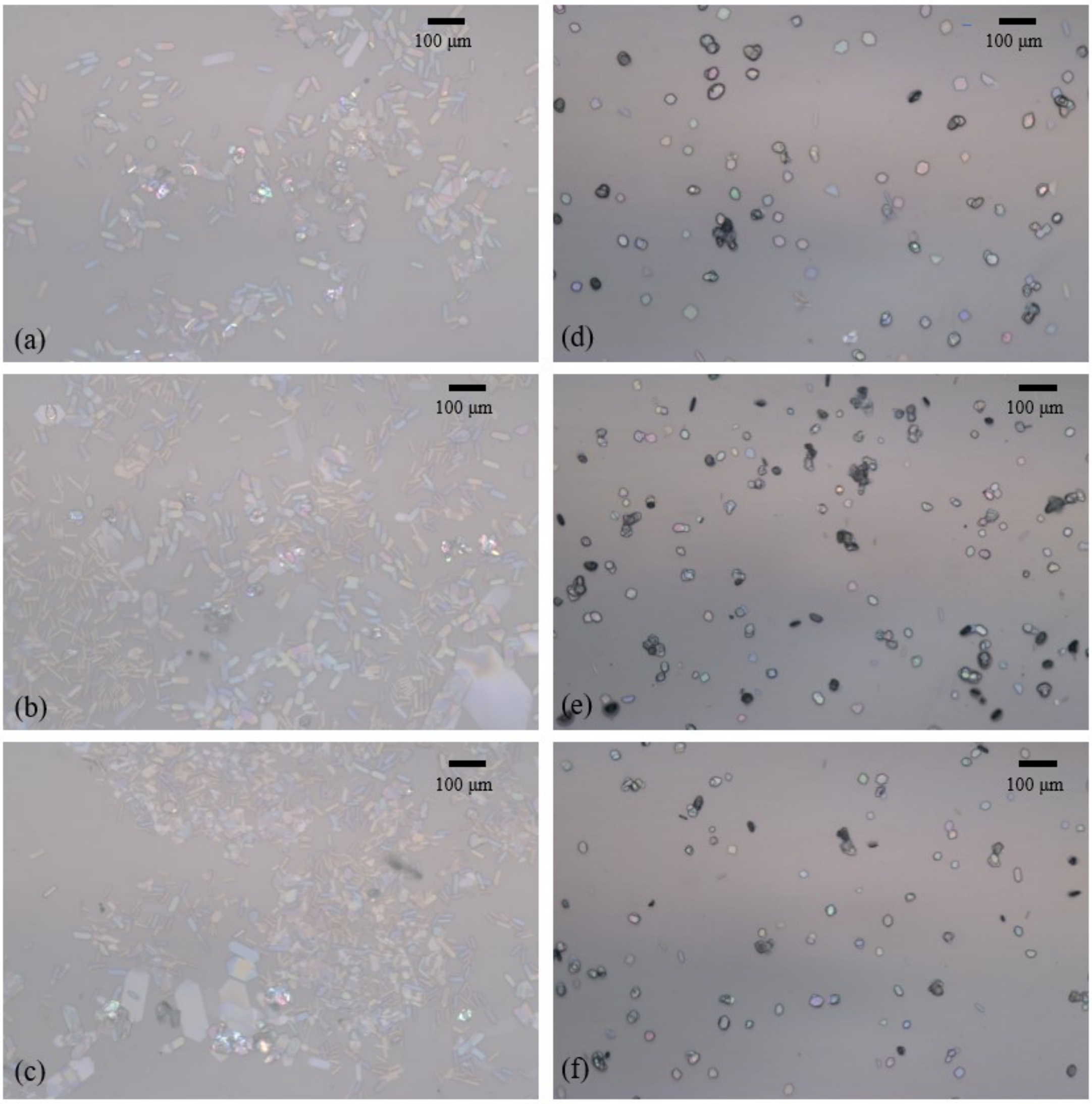

Figure S2 The optical microscopy image of the p-MSB microcrystals. (a-c) the elongated *p*-MSB microcrystals obtained at concentrations of 2 mg per 10 mL CS_2; (d-f) the regular *p*-MSB microcrystals obtained at concentrations of 4 mg per 10 mL CS_2.

S3 The atomic force microscopy (AFM) image

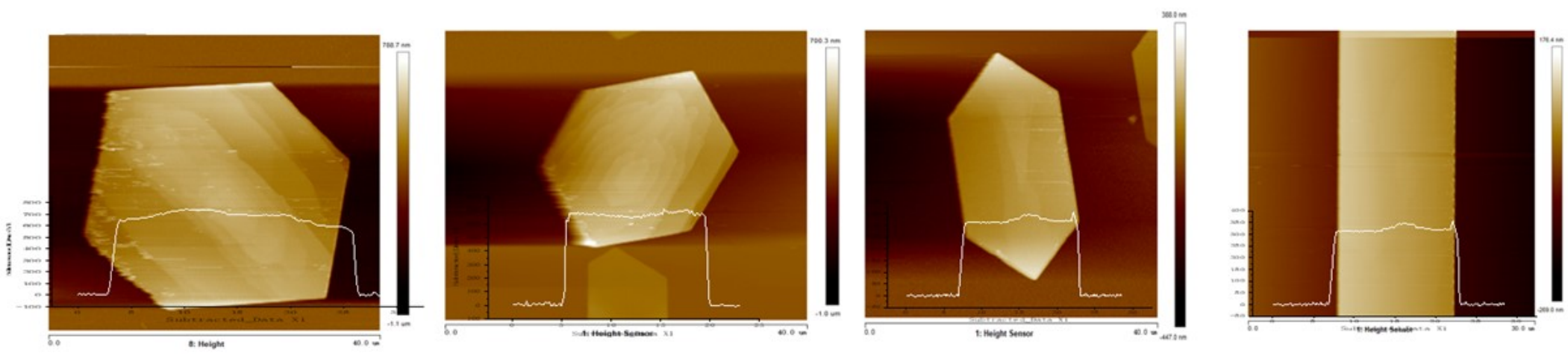

Figure S3 Typical morphology and dimension characterization of microplates. Atomic force microscopy (AFM) and scanning electron microscopy (SEM) reveal smooth surfaces, sharp edges,

and large-scale uniformity. The microplate s exhibit lengths of 10-20 μm, and thicknesses ranging from 400 to 600 nm.

S4 polarization-dependent PL

Figures S4 (a-d) show the molecular and crystal structures of *p*-MSB. The crystallographic data and structure refinement information and photographs is summarized and shown in the Table S1 and Figure S1 and Figure 2 (a). It was orthorhombic with a space group of ***Pbca*** (Z= 4). The *p*-MSB ones have a quasi-planar conformation because of a lack of steric repulsion. This planar structure induces strong supramolecular interactions; this means that the molecules are arranged layer by layer in the well-known herringbone structure, laterally spreading along the ab-plane, and the long c-axis (about 4 nm) is parallel to the crystal z-axis.

Table S1[1]

	p-MSB
empirical formula	$C_{24}H_{22}$
molecular weight	310.44
crystal system	orthorhombic
space group	*P*bca
cell dimensions:	
a, Å	7.362(5)
b, Å	5.883(4)
c, Å	38.95(2)
α, deg	90.000
β, deg	90.000
γ, deg	90.000
V, Å^3	1686.9(18)
T, K	123
Z	4
d_{calcd}, g cm^{-3}	1.222
μ, cm^{-1}	0.686
radiation (λ, Å)	MoKα (0.7107)
2θ range, deg	7.0 – 55.0
crystal size, mm	0.30 × 0.20 × 0.05
no. of reflections measured	16143
no. of unique reflections	1919
no. of variable parameters	120
R (for $F^2 > 2s(F^2)$)[a]	0.1036
wR (for all reflections)[b]	0.1518
goodness of fit, S[c]	0.946

[a] $R = \Sigma||F_o| - |F_c|| / \Sigma|F_o|$.
[b] $_wR = [\Sigma w(F_o^2 - F_c^2)^2 / \Sigma w(F_o^2)^2]^{1/2}$.
[c] $S = [\Sigma[w(F_o^2 - F_c^2)^2] / (N_{ref} - N_{param})]^{1/2}$.
N_{ref} = the number of reflections used in the refinement,
N_{param} = the number of refined parameters.

We measured the polarization-dependent PL Figure S4 (e), showing that the PL intensity oscillates with a period of 180° caused by a direct consequence of the diverse molecular transition dipole moment orientations. The excitation beam from

the femtosecond laser was linearly polarized. A linear polarizer was placed between the laser and the sample, and the excitation polarization angle was varied by rotating this polarizer. It was not deliberately aligned with a specific crystallographic axis because the crystallographic orientation of each individual microcavity was not independently determined during the optical measurements.

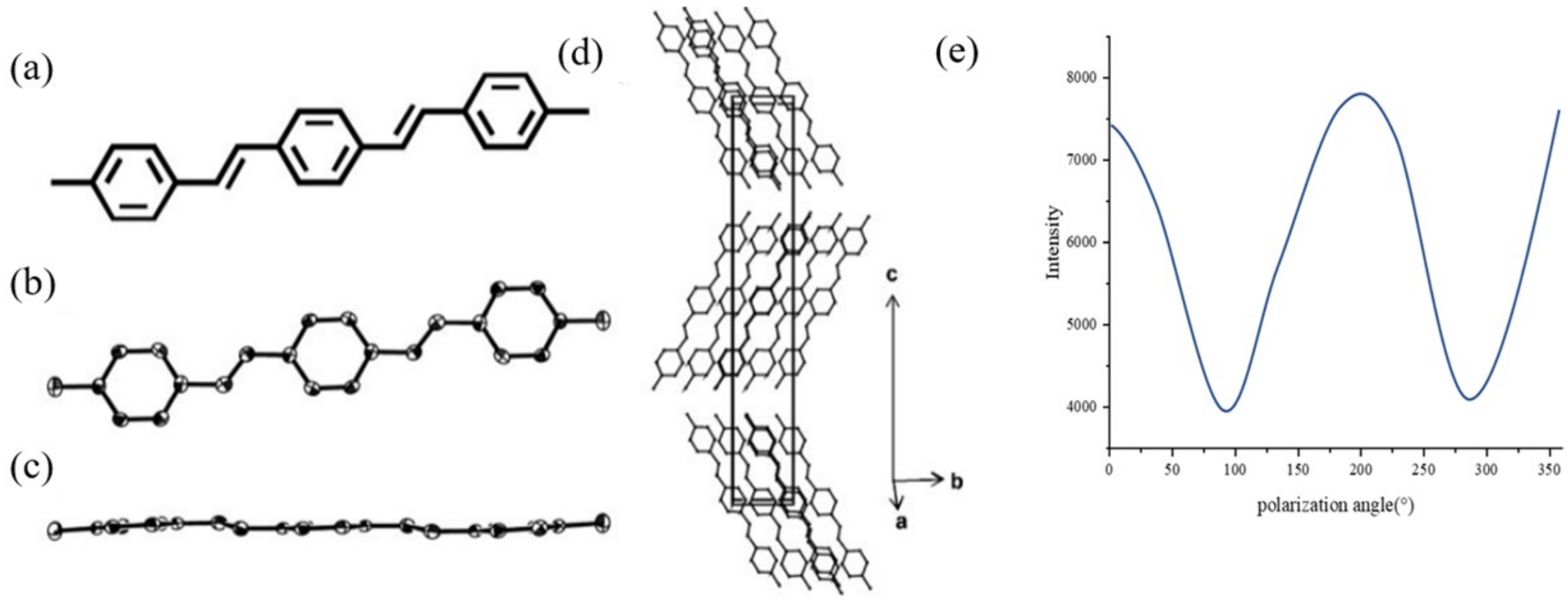

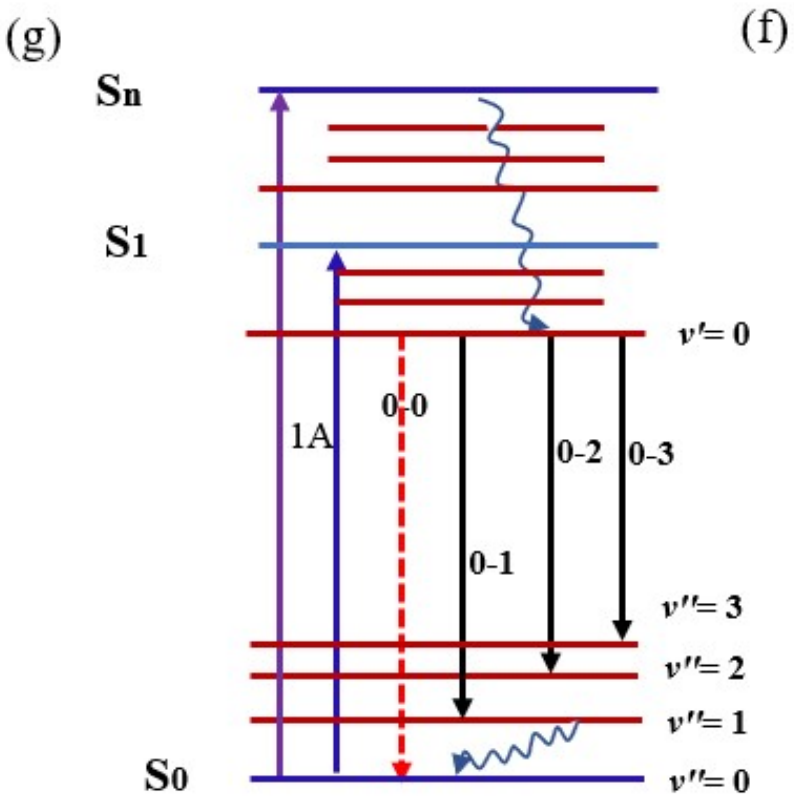

(f)

Photoluminescence (298K)

band	Wavelength (nm)
0-0	~425 nm
0-1	~458 nm
0-2	~488 nm
0-3	~530 nm

Based on the PL spectrum of *p*-MSB single crystal

Figure S4 Chemical structure. (a) ORTEP drawing with thermal ellipsoids at 50% probability level, (b) top view, and (c) side view; (d) crystal packing of *p*-MSB.[1] (e) The dependence of emission intensity on the polarization of excitation light. It was not deliberately aligned with a specific crystallographic axis because the crystallographic orientation of each individual microcavity was not independently determined during the optical measurements. (f) Energy-level diagram for p-MSB H-aggregates. S_0 and S_1 denote the ground and first excited singlet electronic states, respectively, S_1 ($v'=0$) denotes the lowest vibrational level of the S_1 state. The emission bands labeled as 0–1, 0–2, and 0–3 (black solid lines) denote vibronic emission channels from lowest excited state to the vibronic levels of the ground state, respectively. The red dashed arrow indicates the symmetry-forbidden or strongly suppressed 0–0 emission in H-aggregate. Accordingly, the highest-energy component observed in the PL spectrum of an ideal H-aggregate is the 0–1 sideband. (g) The labels 0–1, 0–2, and 0–3 bands and schematic illustration of *p*-MSB vibronic transition based on experimental PL peak distribution.

S5 setup

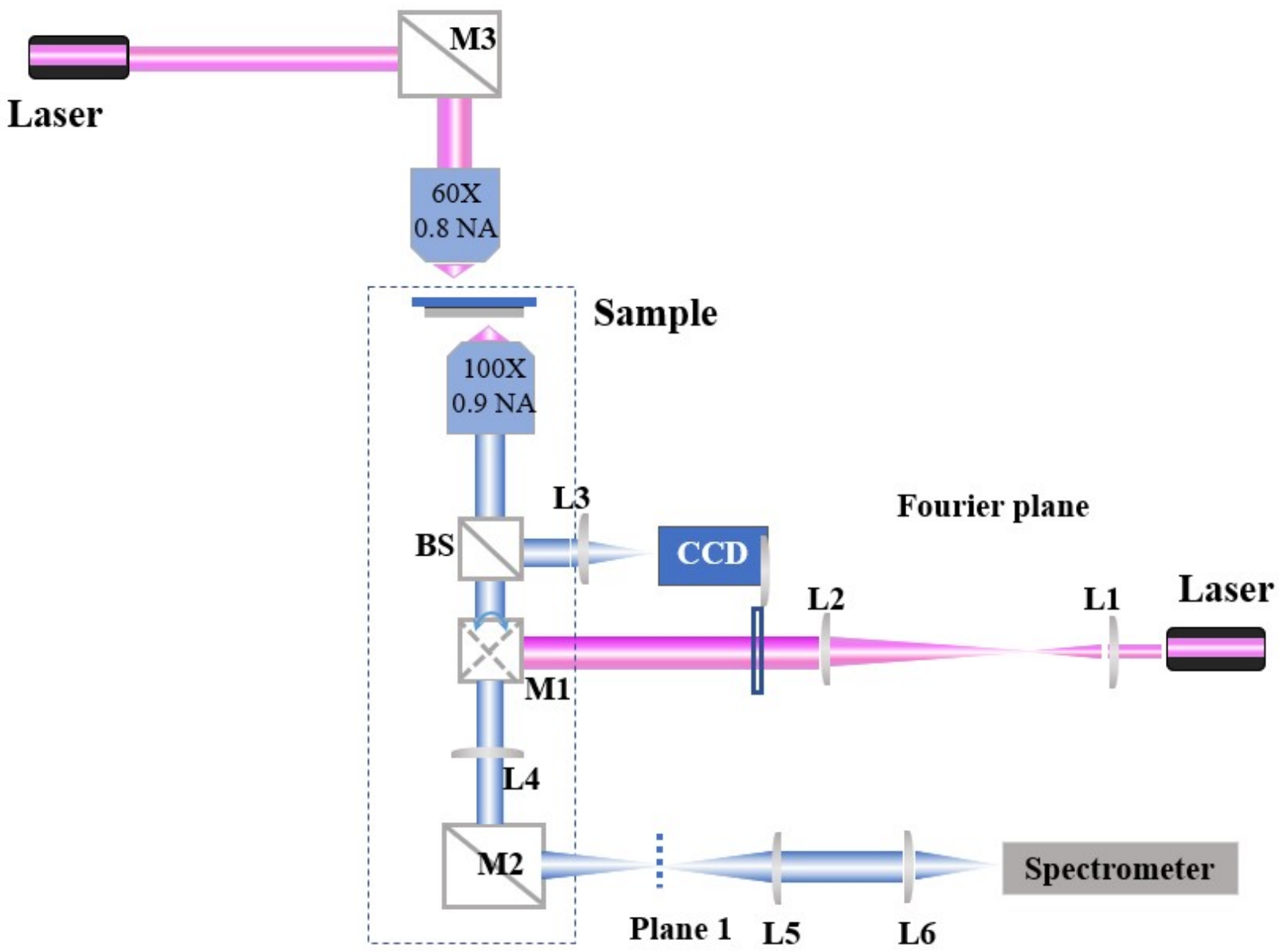

Figure S5 The transmittance optical path for the spatially resolved PL measurements.

Individual microplate was investigated at room temperature in air by a home-made optical microscopy equipped with a 100 × 0.9 NA excitation objective. The second harmonic (400 nm, 150 fs, 1 kHz) of a regenerative amplifier (Spitfire, Spectra Physics) seeded with a mode-locked Ti:sapphire laser (Tsunami, Spectra Physics) was focused to a 50-μm-diameter spot to excite the selected individual microplate. Then PL spectra were collected underneath by using a 100 × 0.9NA objective that was mounted a 3D movable stage. A 450-nm long-wave pass dielectric filter was used to block any scattered excitation light. A linear polarizer was placed between the light source and the sample, and the excitation polarization angle was varied by rotating this polarizer. The detection path did not contain an analyzer. Finally, the collected PL was detected using a liquid-nitrogen-cooled CCD attached to a polychromator (Spectropro-550i, Acton).

S6

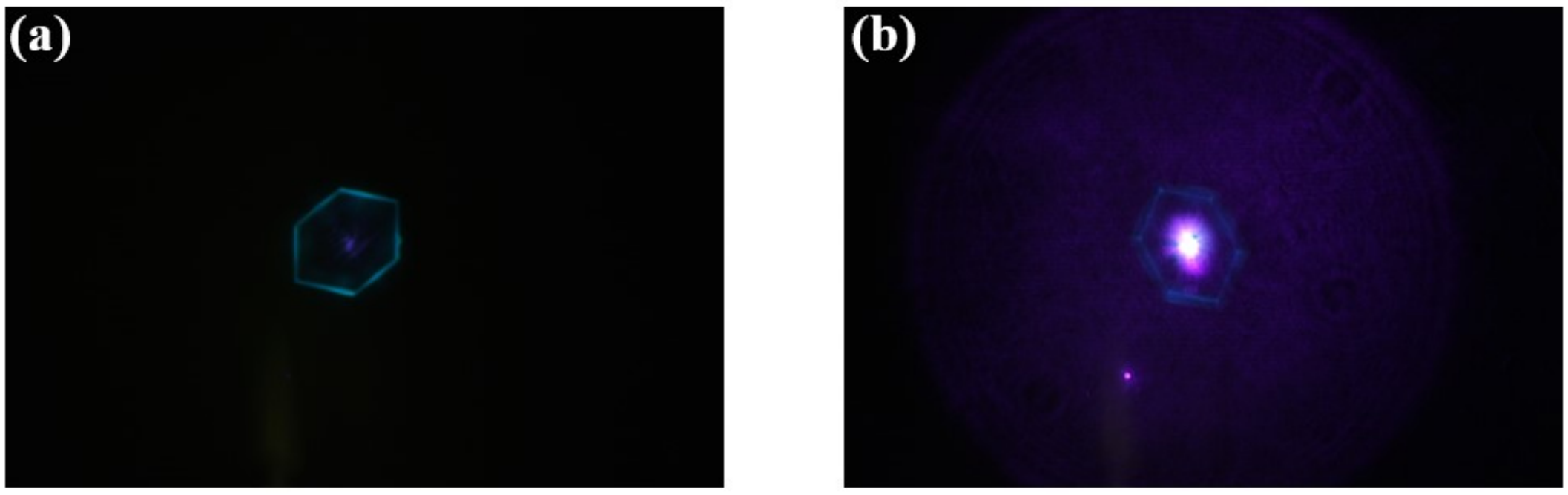

Figure S6 The fluorescence microscopy image with low (a) and high threshold (b).

The threshold of the regular-PHMs can be clearly observed at 4.12 μJ/cm^2. The fluorescence microscopy image with low and high threshold are shown in Figure S6. Below the threshold, the fluorescence intensity is distributed at the edges. Above the threshold, the fluorescence intensity concentrates at the six corners and form lasing.

S7 Decay time curves

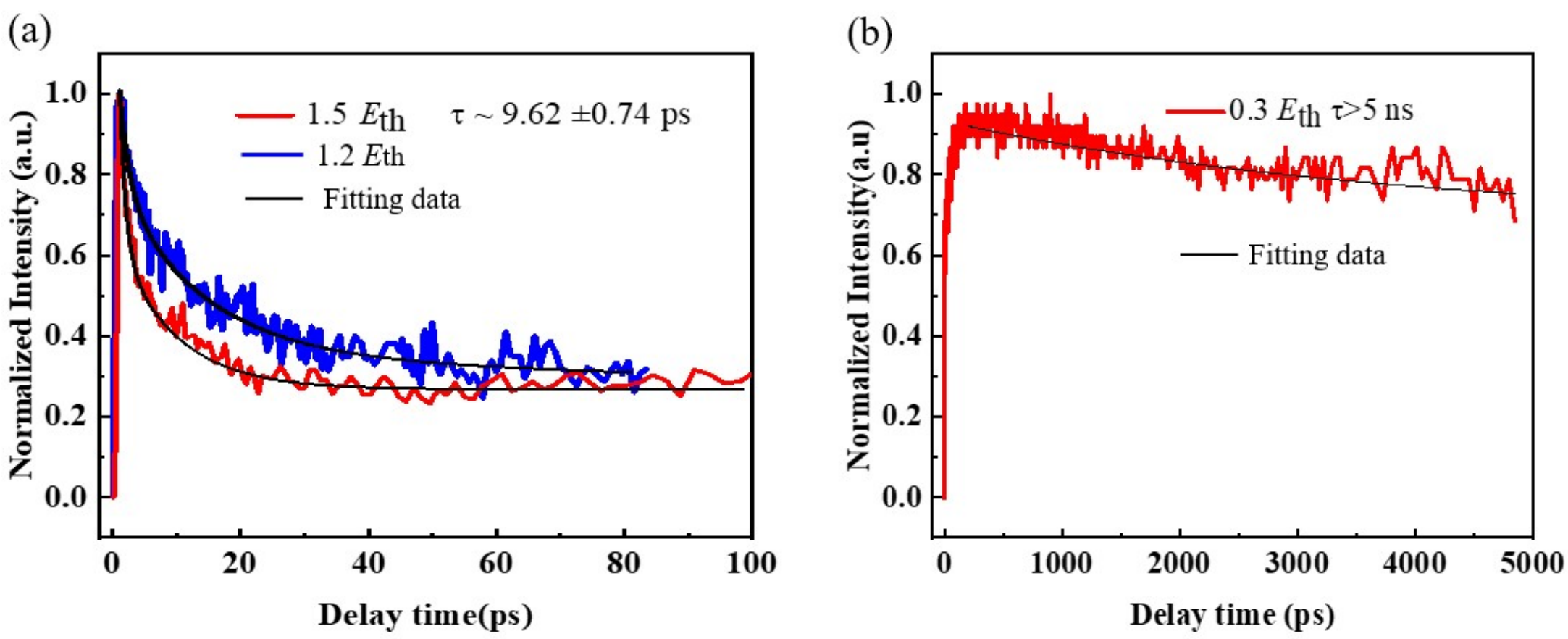

Figure S7 The transient decay time curves of individual regular-PHM (based on Time-Tech Spectra, Co., Ltd,). (a) Above the threshold, decay dynamics curves at 456.6 nm, blue curve: 1.2 E_{th}; red curve: 1.5 E_{th}; black curve: fitting curve. (b) At a very low excitation density of 0.3 E_{th} at 456.6 nm, the decay dynamics curve of individual PHM follows single-exponential function, yielding a decay time > 5 ns.

The decay curve was fitted using a biexponential function and the fitted parameters are: A_1=0.37 ± 0.02, τ_1=0.96 ± 0.11 ps, A_2=0.44 ± 0.02, τ_2=9.62 ± 0.74 ps, R^2=0.989. The decay time always collapses to <10 ps. The fast component τ_1 may be associated with ultrafast exciton depletion under high excitation density. This τ_2 reflects the rapid decay process associated with stimulated emission in the regular p-MSB microcavity, as evidenced by the emergence of sharp lasing peaks and the pronounced narrowing of the emission linewidth.

S8

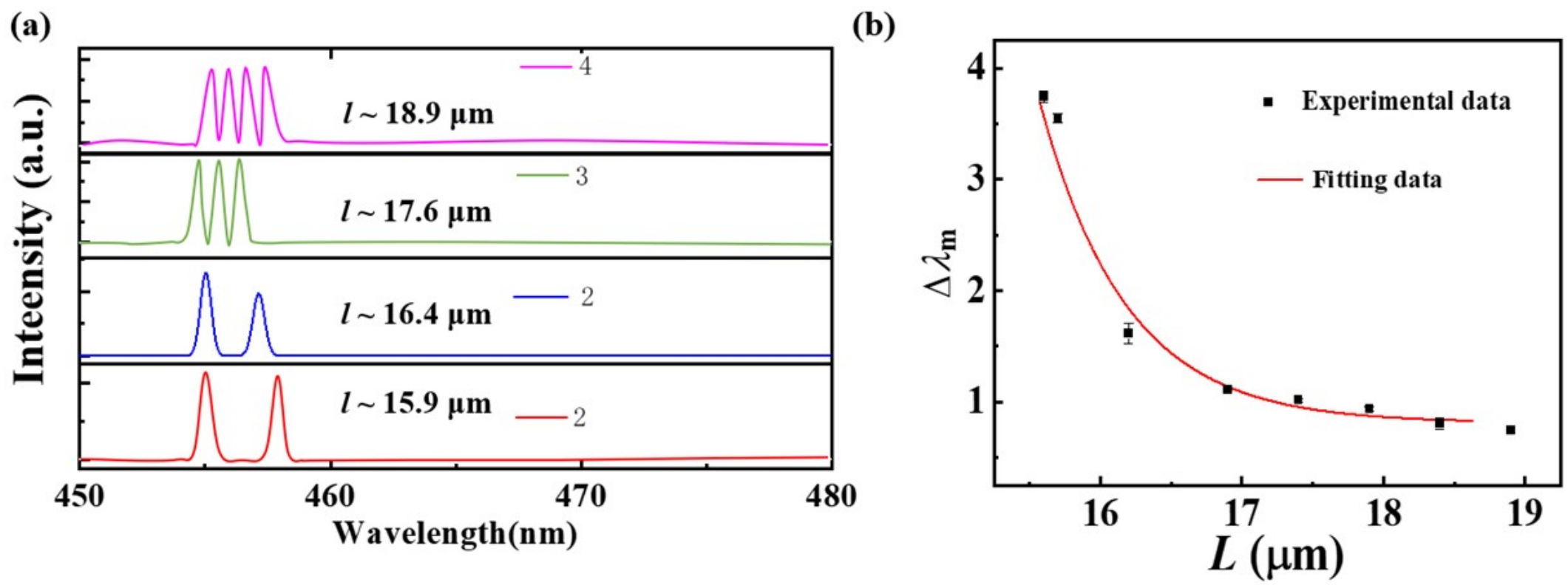

Figure S8 (a) The lasing peak number as a function of side length l were further investigated for the hexagonal cavities. (b) the distance between two adjacent modes ($\Delta\lambda_m$) decreases from 3.71 to 0.72 nm. The extracted $\Delta\lambda_m$ values are inversely proportional to the cavity size, which is consistent with the expected behavior of 6-WGM lasing in hexagonal resonators.

S9

In principle, the hexagonal *p*-MSB microplate can support different optical feedback pathways, supportting a six-bounce whispering-gallery mode (6-WGM), in which light is confined by successive total internal reflections at the six lateral facets of the hexagonal resonator. The experiment data in Figure S8 indicate that the observed optical confinement is more consistent with an in-plane 6-WGM than with a simple FP-type cavity.

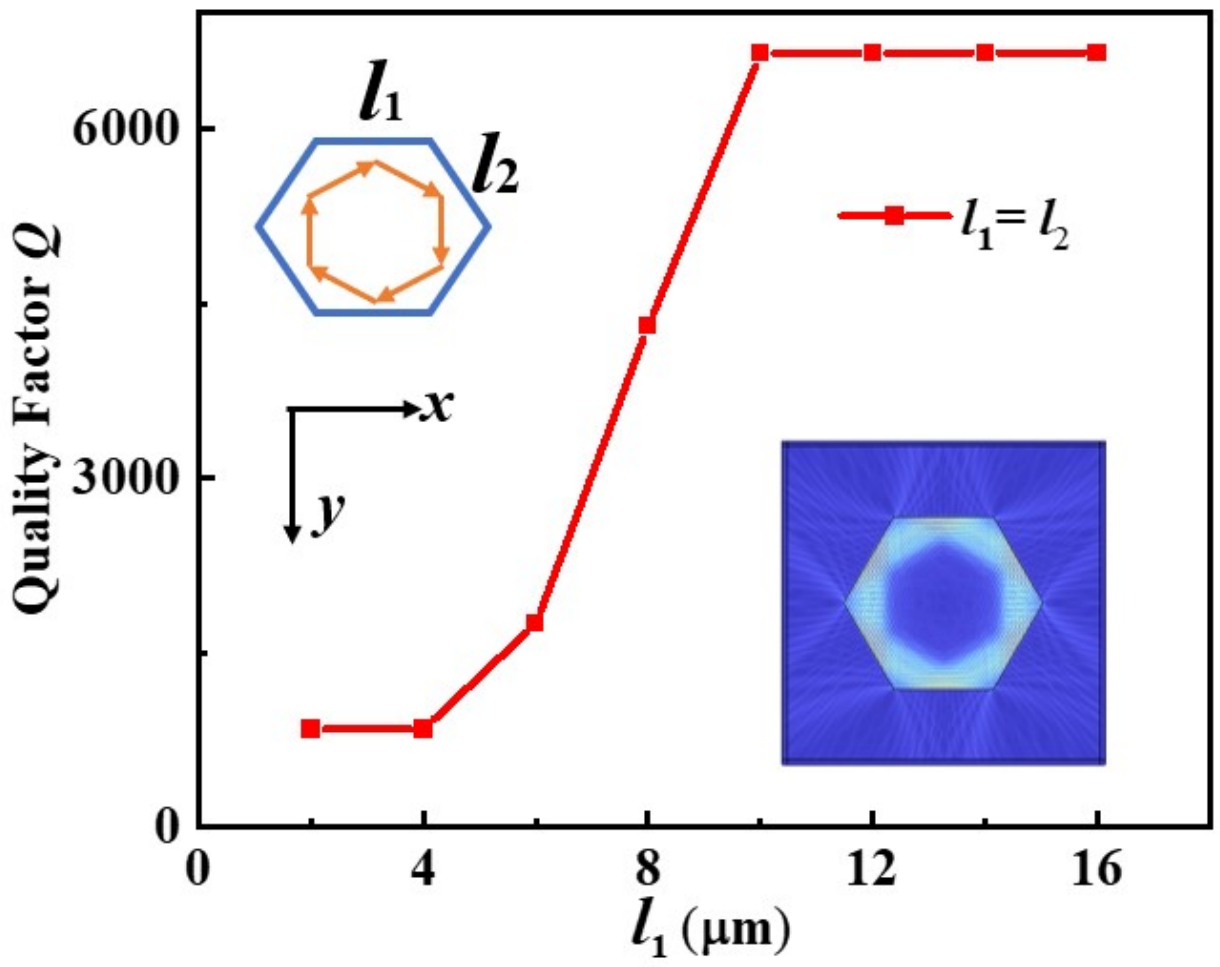

Figure S9: Dependence of the cavity quality factor on the side-length l_1 of the regular-PHMs. The upper-left inset illustrates the schematic illustration of the light path of the six-bounce whispering-gallery mode (6-WGM) in the hexagonal resonator. The lower-right inset shows the corresponding normalized electric-field distribution.

The Q factors in the FEM simulations were obtained from eigenfrequency calculations. The calculated eigenfrequency is complex, $\tilde{f} = f_r + if_i$, where f_r is the resonant frequency and f_i is associated with the radiative decay of the resonant mode. The quality factor was calculated as $Q = \frac{f_r}{2|f_i|}$. In the simulations, the refractive index of the organic microplate was set to n=1.80, and the eigenmodes around λ = 458 nm were calculated. The target resonant mode was selected according to the spectral position near the gain maximum and the spatial electric-field distribution. Only the modes showing strong field confinement inside the microcavity and a 6-WGM closed optical path were used for Q-factor comparison. Because the finite microplate thickness, substrate effects, and out-of-plane radiation losses were not explicitly included, the calculated electric-field distributions and Q factors were used only for qualitative comparison of relative in-plane optical confinement and were not interpreted as absolute quantitative values.

Reference:

(1) Kabe, R.; Nakanotani, H.; Sakanoue, T.; Yahiro, M.; Adachi, C. Effect of molecular morphology on amplified spontaneous emission of bis-styrylbenzene derivatives. *Advanced Materials* 2009, *21* (40), 4034-4038.
